# Pericardiocentesis simulation model: conception and development

**DOI:** 10.1590/acb404725

**Published:** 2025-07-18

**Authors:** Yury Tavares de Lima, Larissa Holanda Gomes, Luiza Matias Marques, Igor Castelo Branco Fontenele Costa, Pedro Henrique Araújo Marques, Josue Viana Castro

**Affiliations:** 1 Universidade de Fortaleza – Núcleo de Biologia Experimental – Fortaleza (CE) – Brazil.

**Keywords:** Education, Medical, Simulation Training, Pericardiocentesis

## Abstract

**Purpose:**

To develop a pericardiocentesis (PCT) simulation model applied for undergraduate medical training.

**Methods:**

A PCT simulator consisted of a torso mannequin, a silicone rubber heart (SRH), a container, and a filling system. The mannequin was submitted to a coronal section and an 18 × 18-cm precordial area opening. The SRH was prepared in accordance with structural dimensions of a normal heart. The elaboration of a structure container to simulate the pericardial cavity consisted of a non-leakable unit inside a cardboard (PcavBox). The PcavBox filling system was connected to a 10-mm diameter tube and a total of 2.5 L of saline solution. This structure was adapted inside the mannequin and covered with a thermoformable rubber material of 2 mm in skin color. For PCT simulation, we used a 10-mL syringe connected to a 14G needle for an imaging guided puncture facilitated by a portable ultrasound.

**Results:**

A SRH was successfully developed and fixed inside the Pcav Box, connected to the fluid pressure system. It was able to simulate a cardiac tamponade scenario identified by ultrasound. A series of 50 punctures was successively performed without liquid leak.

**Conclusion:**

A low-cost PCT simulator was developed and coan be applied to healthcare education.

## Introduction

Despite recent advances in healthcare education, traditional methods of knowledge transfer still predominate in most universities and health schools. Lectures, theoretical assessments, internship periods in primary care units, rotations in hospital wards, and emergency departments are central in undergraduate medical training. However, proficiency acquisition in immediate resolution therapeutic procedures may require specific skills in controlled scenarios and preferably in environments that involve simulation strategies.

Simulation-based education (SBE) is a method that prioritizes the trainee, allowing skills acquisition and graduate development before application in clinical scenarios. This minimizes errors and increases patient safety^1^. Modern simulation platforms were developed to provide realistic experience^2^, but with the limitation of a high-cost technology and simulators.

Patients assisted in the emergency room (ER) are in critical situations that often compromise medical learning in this environment^3^. A very specific scenario is represented by unstable patients present in ER with pericardial pathologies^4^. Among procedures to treat those conditions, pericardiocentesis (pericardial puncture with liquid aspiration–PCT) is the most often performed intervention in critical situations and unstable clinical conditions^5^,^6^.

Most PCTs performed in ERs are due to cardiac tamponade–restricted venous return to the heart^7^. Ultrasound-guided (UG) PCT is the gold standard method for this situation. It reaches a nearly 95% success rate with a low risk of complications, involving approximately 1 to 3% morbidity and a procedure-related mortality below 1%. With the aid of the ultrasound device, the distance between the chest wall and the pericardial fluid can be calculated, the surrounding organs can be seen, and the best positions for inserting the needle during pericardiocentesis can be choose between subxiphoid, apical, and parasternal approaches.

Considering undergraduate medical training in this procedure, PCT simulators are few. The available options seem to have limited application because some are high-cost devices with advanced features and others are simpler to manufacture but uses suboptimal materials^8^,^9^.

Therefore, we sought to develop a simulation device applied for the training of USG PCT in a simulation center of a medical school.

## Methods

This was an experimental study carried out at the Surgical Skills and Simulation Unit of the Nucleus of Experimental Biology, based at the Universidade de Fortaleza, Fortaleza, CE, Brazil. We developed this research to obtain a PCT simulator device in two phases: prototype design with material development, and simulator assembly followed by structural construction and testing.

### Prototype design and development

The PCT prototyping device concept had both external and internal components. The external component was designed using a plastic mannequin torso of adult size (60 × 30 cm). An opening (18 × 18 cm) located in the correspondent precordial area of the torso surface was done.

**Figure 1:**
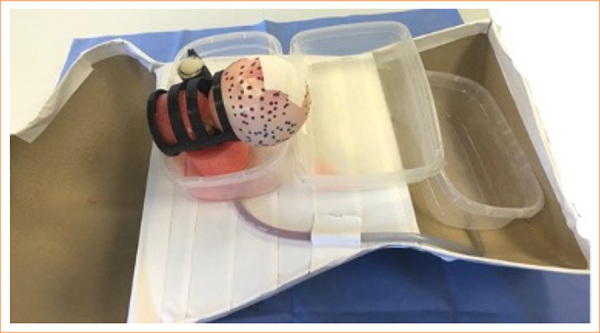
PCavBox structure sketch.

Source: Elaborated by the authors.

The internal component of the prototype consisted of a small rubber ballon filled with 50 mL of saline solution and supported in a plastic and a sponge support in a plastic basin inside the torso plastic mannequin (Fig. 1). The opening was covered with a plate of ethylene vinyl acetate.

### Simulator assembly and structural construction

**Figure 2:**
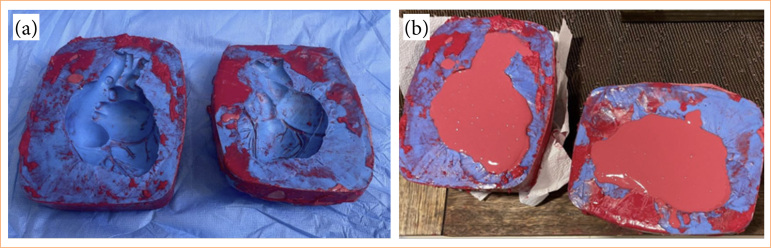
Process of making a life-size heart mold, made of silicone, using an anatomical piece. The still-liquid silicone and the anatomical piece were inserted into a rectangular mold. After drying, the block was cut in half, used as a mold for making a silicone heart.. **(a)** Modeling structure used to elaborate the silicone rubber heart. **(b)** Filled with red Siq Pasta Silbor.

Source: Elaborated by the authors.

The PCT simulator assembly and structural construction followed the prototype 1 with significant differences in external and internal structures. The first step was to reformulate the internal structure, to avoid liquid leakage. In order to obtain a silicone rubber heart (SRH), a silicone rubber modeling was prepared in accordance with structural dimensions of a normal heart. The modeling piece was assembled with siquimol silicone rubber material Siquiplás (Fig. 2a). The internal space of the modeling piece was filled with a dye named Siq Pasta Silbor in the red color in accordance with the proportion recommended by the manufacturer. The amount of 250 mL was prepared to fill the heart modeling structure (Fig. 2b). After the drying process, the halves of the heart mold were brought together and then removed from the modeling device.

The next step was the elaboration of a structure container to simulate the pericardial cavity. To achieve a non-leakable structure, a thermoformable silicone rubber Siq Borflex 30 inside a cardboard (PcavBox) was used. The silicone rubber had to be heated between 180 and 200°C to reach a liquid state and thus be handled.

The PcavBox were 18 × 18 cm^2^ and 6 cm high. The PcavBox structure was divided in two chambers: the lower one, in which the SRH was fixed with a stem, for the elaboration of the side walls and bottom, measuring 18 × 18 cm^2^ and 5 cm high; and the upper counterpart, that compounded the anterior surface and a space between the lower one to promote fluid circulation.

**Figure 3:**
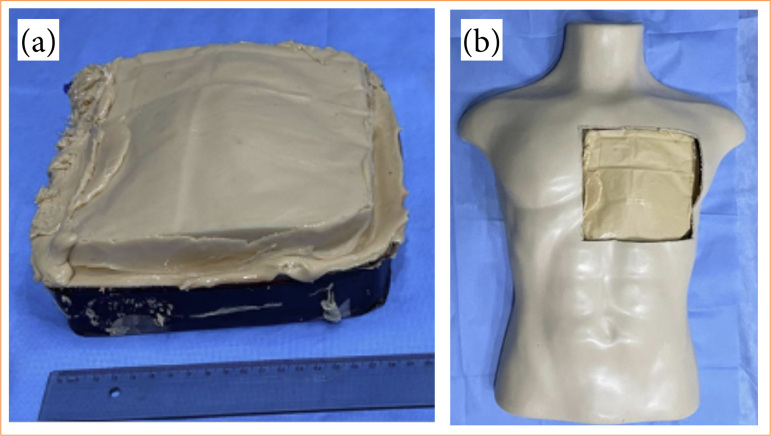
A rectangular mold made from cardboard serves as a template for making the hollow piece that holds the heart and will be filled with liquid, simulating the pericardial space. **(a)** PcavBox final aspect and **(b)** mounted in a mannequin torso.

Source: Elaborated by the authors.

Following that, this system was positioned inside the mannequin with an 18 × 18 cm precordial area opening (Figs. 3a and 3b). The PcavBox filling system was prepared with a 10-mm diameter tube with a 1 m length. The proximal portion of this tube was connected to the container box and the distal portion to a transparent dispenser bottle with 500-mL capacity. With the structure assembled, the silicone mold was filled with saline solution, with a volume of approximately 2.500 mL, enough to cause its slight elevation of the anterior part and fit with the sectional area of the torso. During the filling of the system, the air content inside the system, which accumulates during the process, was carefully removed.

Ultimately, a rubber tissue was prepared to simulate the skin in the shape of the torso. This covering was produced with the silicone rubber pigment Siq Pasta Silbor and used in the color skin no. 4 in the proportions indicated by the manufacturer.

### Testing

Some tests were applied by two of the authors to confirm the final adequacy of the ultrasound-guided pericardiocentesis (UG-PCT) simulator system and the simulation exercise. These included: puncture resistance test, PcavBox system filling test, and UG visualization test.

For puncture, we used a 10-mL syringe connected to a 14G needle. For imaging capture, we used a portable ultrasound system designed for external ultrasound imaging Butterfly iQ+ (Butterfly Network, Inc.). This device was connected to a cellular phone iPhone 14 (Apple Inc.). After applying ultrasound gel on the anterior surface of the square mold, we used the device configuration for the cardiac preset (automatic select of phased array), with depth range set to 15 cm. The imaging was displayed at 6.1-inch (diagonal) all-screen OLED display 2,532-by-1,170-pixel resolution at 460 ppi.

Finally, this research was presented and discussed with the members of the Ethical Committee of the university, especially with the coordinator of the committee. This member is also responsible for the research project development at Nucleus of Experimental Biology. Considering that the study would involve exclusively an instrument/simulator development for only education and training, there were no humans or animals exposed, and at this point the research would not include a validation phase, the project was approved based on Resolution no. 510, of April 7^th^, 2016. Laboratory rules and guidance were respected in all phases of the simulator development. In addition, researchers were always wearing individual protective equipment during laboratory activities.

## Results

**Figure 4:**
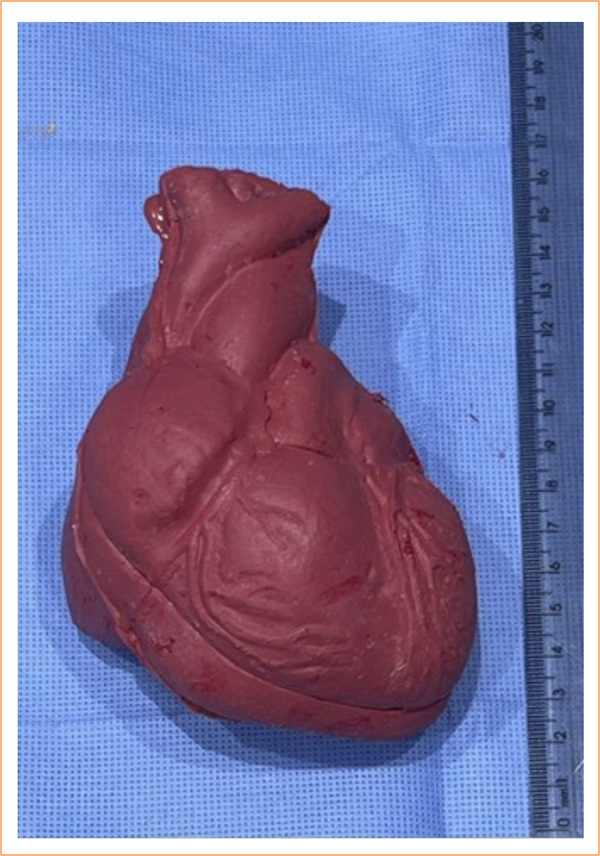
Silicone rubber heart.

Source: Elaborated by the authors.

We have completely transformed a prototype in order to achieve a more adequate system resistant to liquid leakage for PCT simulation exercises. Based on a *modeling structure*, a SRH was successfully developed and obtained in a 14 × 8 × 5 cm with all four chambers, great vessels and coronaries well identified (Fig. 4).

**Figure 5:**
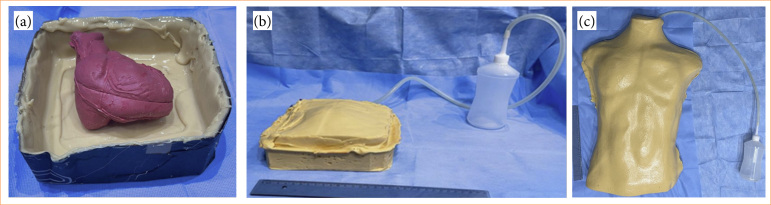
PcavBox assembly process, with one of the halves open demonstrating the positioning of the heart before closing the rectangular piece, until the final appearance of the simulator. **(a)** The silicone rubber heart inside the lower cavity of the PcavBox. **(b)** Hidrostatic PcavBox system. **(c)** Final aspect of the ultrasound-guided pericardiocentesis simulator.

Source: Elaborated by the authors.

In addition, with the SRH fixed in the bottom, a closed structure (PcavBox) was planned and developed to overcome the limitations of creation a pericardial cavity. This structure served as a compartment filled with 2.500 mL of saline solution. The PcavBox was connected to a simple system to maintain the fluid pressure and volume inside despite repetitive puncture. This system consisted of a tube and a reservoir (Figs. 5a and 5b) and was integrated inside of a torso manikin, and the whole structure of the simulator was covered with a thermoformable rubber material and dyed with pigment in the skin color for a human-like appearance. The coverage had 2 mm in thickness to allow appropriate use of ultrasound (Fig. 5c).

To confirm the adequacy of the development of this simulator unit, we performed a sonographic sample and a puncture confirmation.

**Figure 6:**
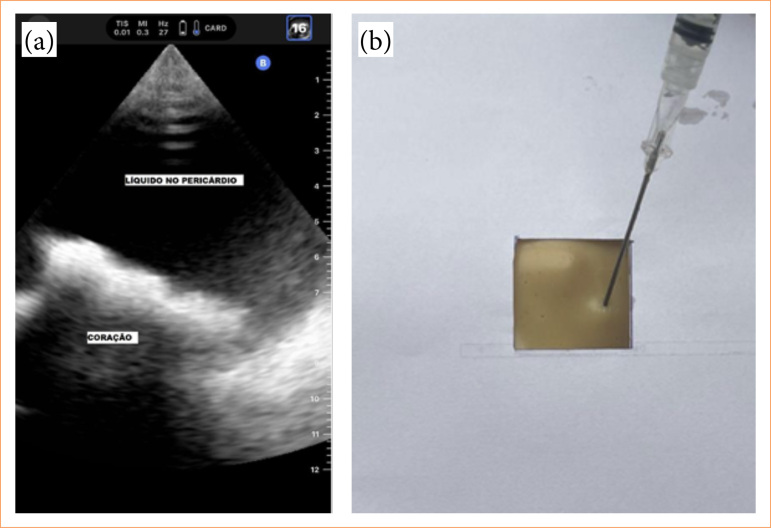
Puncture test of the finished structure, using a portable ultrasound, demonstrating the internal visualization of the assembly, and the external appearance of the puncture area.**(a)** Ultrasound imaging sample, **(b)** puncture site and retrieval of the liquid.

Source: Elaborated by the authors.

The ultrasound generated an image very similar to the image that we obtain from a real patient at the ER resembling a cardiac tamponade scenario, except that for the simulation the heart was not beating. At the puncture site, we were able to retrieve the saline liquid without any further leakage after the procedure (Figs. 6a and 6b).

A total of 50 PCT simulation exercises were done by two of the authors with expertise in this type of procedure, and the impression was that the resistance directing the needle up to the correct spot to retrieve the liquid was compared with human tissues. In addition, after the punctures, we did not find any leakage of fluid through the Pcav system.

The assembly of the entire simulator, from preparing the materials, handling the silicone rubbers that require heating or mixing with a catalyst, drying the materials and finishing the assembly of the parts, took around two days, since the rubber of thermoformable silicone needs to dry satisfactorily for 24 hours to be handled without risk of structural damages.

## Discussion

SBE has been an important strategy in healthcare professional education. It has become essential in most scenarios, especially those which involve prompt actions as in ERs. It requires prompt decision-making and carrying out procedures in a timely manner. Error is human and can cause disabilities or even death^10^. Furthermore, ER training of complex procedures for trainees is rare and depends on a random chance of exposure^11^. Thus, more realistic and accurate models are recommended to promote safe learning and consequently maximize patient safety and minimize risks^4^.

PCT is an uncommon procedure in clinical practice. Historically, it has been associated with high mortality due to critical situations such as clinical decompensation and cardiac tamponade. Among the complications, heart perforation and pneumothorax can be fatal. UG-PCT is considered the gold standard for reducing complications and guide the procedure^12^. It is reported that emergency or intensive care physicians face the problem of performing the procedure without experience, especially when an image-guided one is needed^13^.

Considering that, SBE is necessary to encourage proficiency and competence in performing PCT^14^. Especially when introduced at the undergraduate medical training scenario. It should be noted that the simulators available are few and usually a high-cost device. It limits their acquisition by institutions in low-income countries^13^,^15^.

The type of simulator developed in the present study is configured as a task trainer. It consisted of a unit that seems to represent safe for high-volume basic skills training or even though a device useful to train key elements of UG-PCT^16^. Barnes and Paterson-Brown^17^ pointed out the main advantages of this type of simulator, including low cost, ease handling and repetition of practice. On the other hand, the need to replace the skin or parts of the structure due to the high use and focus area limited to the region of the task trainer could be seen as limitation.

Two favorable points of the model developed by our group are its low cost and high applicability, including between undergraduate students. These are important aspects as they allow progressive improvement of trainees, especially when applied in developing countries^17^.

It is important to say that it will be necessary for the development of this initial model to improve the fidelity of the device, as well as its validation and classification. It is noteworthy that the term fidelity is related to the realism of the learning experience^18^.

For the development of expertise in performing procedures, a three-dimensional (3D) understanding of the patient’s anatomy is necessary. This is obtained with the sequential steps and when appropriate instruments for each step is generated, facilitating the development of psychomotor skills associated with the procedure^16^. We feel that the option presented here seems to be simple, but it incorporates the possibility of an image-guided puncture as a first step in order to obtain the competence.

A study promoted in the United States of America was based on the development of a simulator for pericardiocentesis from 3D printing. The development of the simulator initiated, as ours, by the generation and 3D printing of the heart molds. At the end, the fidelity and echogenicity of the simulator were considered satisfactory^19^. Preliminary evaluation suggests that this model is more straightforward to be implemented. The future application for undergraduate students training can be obtained with certain degree of fidelity.

Scholars created a low-cost PCT model using pig skin and ribs, gelatin, plastic bag, and avocado. The limitation of this type of simulator is the use of biological tissues^20^. In comparison with our model, we emphasize that non-biological models facilitate its application, especially in low-fidelity simulators.

Researchers in Singapore have developed an agar-based model of PCT, using agar jelly as the ultrasound medium to simulate human tissues. This model focused on the aspiration of fluid inside the flask but did not include the insertion of a drain due to the friable nature of the agar jelly when drains with a larger diameter are used. It is noteworthy that the lower quality of ultrasound images in the agar-based model may be related to the increase in irreversible artifacts and distortion of ultrasound images with each new attempt to perform the procedure. Our model was capable of generating excellent images, despite of the non-beating SRH.

Researchers have created a very low-cost portable alternative model for UG-PCT, using a gelatin frame, golf ball, and balloon, with the gelatin frame connected to a frame simulating the pericardium and myocardium. This model does not have external anatomical references to guide the placement of the transducer and the needle, nor does it offer simulated physiological parameters to evaluate the success of the procedure^21^.

A survey carried out in the United States of America used an economical 3D cardiac model, simple to assemble, with anatomical precision, and high fidelity for PCT training. The simulator consisted of a latex balloon-in-balloon pericardium filled with colored saline and placed in an ultrasound-compatible gelatin mold. The pericardium was crafted by hand, with cutting and gluing of latex balloons around the cardiac model. This model is like the initial prototype used by scientific initiation students^22^.

North American researchers built a model for low-cost PCT using a ping-pong ball filled with red dye (simulating the right ventricle) and a 250-mL normal saline bag simulating the stroke, wrapped in an artificial ribcage, and kept in the place by wax gel. For model operation, the internal saline bag was connected to a 1-L saline bag outside the assembly to be a fluid reservoir for repeated uses. The construction of the model had an estimated cost of $ 200 and lasted from 16 to 20 hours, with the longest time spent cooling the gel wax. Subsequently, the model was submitted to simulation-based testing by 23 residents and medical students, with a satisfactory evaluation regarding realism, aid in the recognition of important structures (pericardium, heart and pericardial sac), being well evaluated for repeated use over a period of a year^23^.

A study carried out in Germany developed a low-cost PCT model based on the following materials: polyvinyl chloride (PCV) addition polymer boards (22 × 22 × 14 cm, 0.3-cm thick) filled with wax gel until reaching the liquid state; a celluloid table tennis ball to simulate the heart filled with water from a syringe; and a balloon for placement of the ball simulating the heart with red water. The flask was placed over the solidified gel wax. The container was then filled to the brim with ultrasound gel, and any air bubbles within the ultrasound gel were smoothed out with a spoon or syringe. Finally, the model was covered with a silicone structure like an elastic band normally used for gymnastic exercises. The materials cost an average of 60 euros, and the preparation time was 2 hours. The model was later tested by 67 participants (specialists, residents, and medical students), withstanding 60 punctures for four weeks. In addition, it was well evaluated regarding the visibility of the effusion and its discrimination from the surrounding tissues, increasing confidence in performing the procedure, despite being considered unrealistic^13^.

Our impression is that these options are not superior to our simulator model when considering fidelity, and especially the characteristics observed in the construction of molds based on gel, wax or gelatin, that require several hours of dedication for their production. A study conducted in the United States of America to determine the feasibility of a limited-use and low-cost UG-PCT model as a training tool for emergency physicians succeeded in creating a practical model not dependent on gel wax or mold-based gelatin, easy to implement and acceptable for training in the procedure. The model consisted of two latex balloons, sterile surgical lubricating gel, Tegaderm transparent film dressing, psyllium fiber supplement Metamucil, a 7-L disposable plastic basin, food coloring, plastic wrap, and tap water^9^. In this example, the final aesthetic result allowed us to classify it as low fidelity, not having clear anatomical references for a realistic procedure, in the external aspect of the simulator.

Thus, the development of the simulator model presented in this work has the advantage of being produced in non-organic material, which gives greater durability to the parts. This material selected to assemble is inexpensive, accessible and ease to handle. These characteristics could provide, after validation, a wide application for repeated training at any level. Our model incorporates the possibility of using an image guided procedure in the three variations of techniques used for PCT.

As for the limitations identified in our UG-PCT simulator, we highlight the necessity for validation, as well as the evaluation of its long-term durability and effectiveness in the clinical learning of the procedure. The viability of commercializing the simulator for its dissemination in local and regional simulation centers would initially require an awareness of its importance and necessity on the part of educators and by the trainees themselves. Regarding the production of the simulator and the handling of its materials, during the heating of the thermo-moldable silicone rubber, the substance releases smoke in some proportion, in addition to having the risk of managing this material to a temperature higher than that recommended by the manufacturer. At this point, the use of individual protection and safety equipment for handling chemical products in a controlled environment is necessary. In addition, a greater number of simulation exercises will be necessary to identify the real capacity of the system to define the exact number of punctures that the PcavBox is able to be submitted without compromising the simulation performance. Despite the aim of this study did not include a validation test, it is our intention to do so in the future.

## Conclusion

Given the current scenario, in which the availability of simulators for PCT are rare or even inexistent, the incorporation of a low-cost and medium-fidelity simulator becomes an important scientific apparatus for SBE involving undergraduate medical students, as well as health professionals.

The UG-PCT simulator presented here seems to be an important alternative as it is a low-cost development and can be widely applied in undergraduate and postgraduate training.

It is the intention of our group to develop a research to validate this initial model and maybe apply it for local and regional education involving PCT.

## Data Availability

All data sets were generated or analyzed in the current study.
